# Update and review of the multidisciplinary management of stage IV colorectal cancer with liver metastases

**DOI:** 10.1186/1477-7819-7-72

**Published:** 2009-09-29

**Authors:** Sherif Raafat Zikry Abdel-Misih, Carl R Schmidt, Paul Mark Bloomston

**Affiliations:** 1 The Ohio State University/James Cancer Hospital, Columbus, Ohio 43210, USA

## Abstract

**Background:**

The management of stage IV colorectal cancer with liver metastases has historically involved a multidisciplinary approach. In the last several decades, there have been great strides made in the therapeutic options available to treat these patients with advancements in medical, surgical, locoregional and adjunctive therapies available to patients with colorectal liver metastases(CLM). As a result, there have been improvements in patient care and survival. Naturally, the management of CLM has become increasingly complex in coordinating the various aspects of care in order to optimize patient outcomes.

**Review:**

A review of historical and up to date literature was undertaken utilizing Medline/PubMed to examine relevant topics of interest in patients with CLM including criterion for resectability, technical/surgical considerations, chemotherapy, adjunctive and locoregional therapies. This review explores the various disciplines and modalities to provide current perspectives on the various options of care for patients with CLM.

**Conclusion:**

Improvements in modern day chemotherapy as allowed clinicians to pursue a more aggressive surgical approach in the management of stage IV colorectal cancer with CLM. Additionally, locoregional and adjunctive therapies has expanded the armamentarium of treatment options available. As a result, the management of patients with CLM requires a comprehensive, multidisciplinary approach utilizing various modalities and a more aggressive approach may now be pursued in patients with stage IV colorectal cancer with CLM to achieve optimal outcomes.

## Introduction

Colorectal cancer(CRC) is the third most common noncutaneous malignancy in both men and women(Men - Lung, Prostate; Women - Lung, Breast) [1]. Approximately 150,000 cases of colorectal cancers are diagnosed annually in the United States, 25% of which present with liver metastases [2]. In total, up to one-half will develop liver metastases. The management of colorectal liver metastases (CLM) has changed dramatically in the last decade as a result of significant improvements in both medical and interventional therapies now offered. With improvements in therapy come increasing challenges to surgeons and oncologists as to the optimal management of CLM.

Patients with CLM require a comprehensive multimodality treatment approach, but surgical resection does remain the mainstay of curative therapy. In the era of 5-Fluorouracil, achievement of ten-year survivals of 17-25% after attempted curative hepatectomy were encouraging [3]. However, the importance of surgical intervention for curative therapy was particularly evident in a study by Scheele et al. demonstrating improved survival following hepatectomy for CLM compared to patients with unresectable disease and to patients with resectable disease that did not undergo operation [4]. In the group undergoing potentially curative resection, five- and ten-year actuarial survival of 40% and 27% were achieved, respectively. In contrast, median survivals of 6.9 months and 14.2 months without any five-year survivors were seen in the unresectable group and the nonoperative group with resectable disease, respectively. Unfortunately, only 10-20% of patients with colorectal liver metastases are actually resection candidates. However, of those patients amenable to resection, five-year survivals of over 50% are possible, despite recurrences being common [5-7].

Advances in systemic chemotherapy and targeted biologic therapy are occurring at a rapid rate with multiple trials demonstrating encouraging results compared to historical data with increased median survivals of approximately 20 months for patients with unresectable disease [8,9]. However, a thorough discussion of completed and ongoing trials for chemotherapy is beyond the scope of this review. Herein, we will focus on the surgical aspects of the management of CLM and restrict discussion of chemotherapeutic agents to the context of their use in conjunction with surgical management.

## Assessment of Resectability

### Patient selection

Traditional dogma governing surgical intervention restricted hepatectomy for colorectal liver metastases to patients with unilobar disease, less than four lesions, lesions less than five centimeters in greatest dimension, and those without extrahepatic disease. However, with improvement in surgical techniques and advancements in systemic therapy using a multidisciplinary approach, focus has shifted towards the amount of residual liver after resection, or the future liver remnant (FLR). As such, tumor-related factors are no longer considered absolute contraindications to surgical resection, although they are still harbingers of more aggressive tumor biology [10-12]. Historically, these characteristics of the primary tumor and CLM have been utilized to determine the risk of recurrence after curative hepatectomy.

Fong et al. examined this concept closely and created a clinical risk score(CRS) using regression analysis in examining multiple clinical factors of 1001 patients that underwent hepatectomy for CLM [13]. Fong et al. found five clinical criteria that were prognostic for patient outcome. These included node-positive primary, carcinoembryonic antigen(CEA) greater than 200 ng/mL, greater than one liver lesion, any lesion greater than five centimeters, and disease free interval less than one year from resection of the primary lesion. These five clinical criteria were implemented into a CRS which may be utilized preoperatively as a prognostic indicator of long-term outcome and hence aid in patient selection.

In 2006, the American Hepato-Pancreato-Biliary Association (AHPBA), Society of Surgical Oncology (SSO), and Society for Surgery of Alimentary Tract (SSAT) convened for a consensus conference to examine many of the issues regarding indications for hepatectomy for CLM [14]. Recommendations put forth by this panel focused on the ability to obtain margin-negative resection while leaving a FLR consisting of at least two contiguous hepatic sectors, adequate inflow, outflow, biliary drainage, and a greater than 20% FLR of liver volume in a healthy liver.

### Impact of Margin Status

The importance of margin status after resection has been discussed and studied in which multiple studies have demonstrated improved disease-free and overall survival(OS) in patients who underwent margin negative resections. Choti et al. demonstrated that those patients with positive margin resection had survival of 24 months versus 46 months in those with negative margins [15]. Likewise, Pawlik et al. demonstrated decreased OS with higher local recurrence rates for those with positive margin resection [7]. Interestingly, in a subgroup analysis, Pawlik's study demonstrated that the previously thought resection margins of at least one centimeter did not demonstrate a statistically significant difference in recurrence rate, site of recurrence, or OS relative to those patients with close (i.e. 1 - 4 mm) margins. This led the SSO consensus group to conclude that, while wide margins of least one centimeter should be sought, anticipation of a close margin should not preclude resection [14].

Interestingly, with the improvement in response rates with modern day chemotherapy, recent studies are reexamining the requirement of margin negative resection to achieve improved outcomes. Recently, de Haas et al. examined 436 patients of which 234 underwent R0 resection while 202 patients underwent R1 resection [16]. The R1 resection group, not unexpectedly had a higher number and size of CLM often with bilobar disease which made safe negative margin resections prohibitive. Interestingly, the five-year OS rate was 60% and 57% for R0 and R1 resection, respectively(p = 0.27). Five-year disease free survival(DFS) was 29% and 20% for R0 and R1 resection, respectively(p = 0.12). However, when examining intrahepatic recurrence, a significant difference was observed with a higher recurrence of 28% associated with R1 resection versus 17% for R0 resection.(p = 0.004) This study also determined poor independent predictors for OS included CEA>10 ng/ml and major hepatectomy. Factors that were poor predictors for positive margin resection included intraoperative blood transfusions, bilobar disease, and CLM > 3 cm. Therefore, it appears that R1 resection is associated with a higher recurrence rate, however, with improving chemotherapy, survival with R1 resection appears similar to R0 resection and may substantiate an aggressive surgical approach to treatment of CLM even with questionable ability to achieve margin-negative resection and may no longer be an absolute contraindication to attempted curative resection in highly selected patients.

### Extrahepatic Disease

The presence of extrahepatic disease (EHD) has traditionally been thought of as an absolute contraindication to hepatectomy for CLM, however there is increasing discussion in the literature questioning this. As illustrated in Table 1, over the last two decades, there have been multiple studies demonstrating reasonable survival rates in selected patients with EHD that were treated with an aggressive surgical approach. Additionally, studies have examined the question of EHD as a whole and in terms of sites of EHD specifically(e.g. pulmonary, portal adenopathy, peritoneal) [11,17-19]. There is increasing support arguing for a paradigm shift from an absolute contraindication to hepatectomy in the presence of EHD to use of hepatectomy combined with resection of EHD in highly selected patients.

**Table 1:** Five-year Overall Survival of patients with EHD undergoing hepatic resection for CLM.

**Study**	**Year**	**# of Patients**	**Patients with EHD(% of total)**	**5 year Overall Survival of patients with EHD(%)**
Scheele et al [11].	1995	469	47(10)	26

Fong et al [13].	1999	1001	43(4)	18

Minagawa et al [97].	2000	235	17(7)	21

Elias et al [18].	2003	376	111(29)	20

Elias et al [19].	2005	308	84(27)	28

Carpizo et al [20].	2009	1369	127(9.3)	26

Six large studies in the past two decades that examined the significance of EHD and the overall 5-year survivals in patients with CLM that underwent hepatic resection.

Elias et al. retrospectively examined 376 patients who underwent hepatectomy for CLM, of which 111 (30%) underwent resection of various foci of EHD [18]. While five-year OS between patients without and with EHD was 34% and 20%, respectively, outcome was dependent upon the distribution of EHD as well as the complete resection of all EHD. Of the 111 patients with EHD, an R0 resection was achieved in 77 (69%) while 34 (31%) had incomplete (i.e. R1 or R2) resection. When complete (i.e. R0) resection was possible, five-year survival was 29% in those with EHD compared to 38% for those without EHD(p = 0.072). These results when compared to those patients who historically received chemotherapy alone demonstrated more favorable outcomes [18].

In a retrospective study by Carpizo et al., 1369 patients with CLM that underwent resection were examined, 127 of which had concurrent EHD resected at the time of hepatectomy [20]. Patients with EHD had worse three- and five-year survival rates(47% and 26%, respectively) compared to those patients without EHD(67% and 49%, respectively)(p < 0.001). Additionally, multivariate analysis revealed four factors that were independently associated with worse survival including CRS ≥ 3, EHD detected intraoperatively, incomplete resection of EHD, and neoadjuvant chemotherapy. However, recurrence in the EHD group was almost inevitable seen in 110/116(95%) patients.

Another topic of consideration with relation to EHD that continues to be discussed is extrahepatic lymph node disease. Regional lymph node disease traditionally has been thought to be a poor prognostic indicator with relation to patient outcome and considered a contraindication to liver resection. More recently, this has been questioned and Adam et al. recently examined the results of 763 patients who received preoperative chemotherapy of which 47 then underwent hepatic metastasectomy with simultaneous lymphadenectomy [21]. Five-year OS of 11% and 23% were seen in patients with lymph node involvement and without lymph node involvement(p = 0.004), respectively. Of particular importance on further analysis was lymph node location. Observed five-year OS was 25% for hepatic pedicle nodes versus 0% for celiac and para-aortic lymph nodes(p = 0.001). Multivariate analysis determined that celiac node involvement and age ≥ 40 were independent poor prognostic indicators. Adam et al. concluded that in well selected patients with disease responsive to chemotherapy, simultaneous hepatic resection with pedicular lymphadenectomy is reasonable. Similarly, Oussoultzoglou et al. examined 45 patients with CLM and pathologically proven hepatic lymph nodes [22]. In their analysis, the node location was divided into 2 areas. Area 1 comprised proximal adenopathy within the hepatoduodenal ligament and retroduodenopancreatic zones. Area 2 was comprised of distal adenopathy involving common hepatic artery and celiac axis. Overall 3-year and 5-year survival rates were 29.7% and 17%, respectively. In sub-analysis, area 1 median 3-year and 5-year survivals were 34.3% and 25.7%, respectively versus 30.1% and 16.7% for area 2(p = 0.7755). This study supports the concept that with modern day chemotherapy, it may be beneficial to pursue an aggressive curative approach and to extirpate sites of hepatic disease and EHD including lymph nodes. Additional support for an aggressive approach in patients with distant, resectable EHD has also been demonstrated by de Haas et al. in which they pursued sequential resection of various distant disease sites with reasonable outcomes with five-year survivals over 30% [23].

Hence, in patients with good response to chemotherapy, it is reasonable in highly selected patients to pursue a safe, aggressive surgical approach to extirpate various sites of disease inclusive of regional lymph nodes and sites of distant, resectable disease.

### Multifocal Disease

Early experiences with resection for CLM reported the presence of greater than four lesions as a harbinger of poor outcome and a contraindication to resection [10,24,25]. These early reports were often confounded by poor surgical outcomes for more complex operations. For some time, the cutoff for resection was held at four lesions, but recently, larger centers have begun to report positive experiences with resection of multiple lesions and this dogma may not hold true as surgical technique and adjuncts improve. Pawlik et al. reviewed 159 patients with four or more CLM (median 5, range 4-14) who underwent curative resection with median survival of 62 months and a five-year survival of 51% [26]. As well, Kornprat et al. reported median survival of 44 months and five-year survival of 33% in 98 patients with resection of four or more (median 5, range 4-15) CLM [27]. These results lend support that the number of CLM should no longer be an absolute contraindication and a more aggressive approach may be taken in selected patients.

### Bilobar Disease

The presence of bilobar disease is traditionally one of the most concerning characteristics in patients with CLM being considered for hepatectomy. However, this concern has not been substantiated with data. In fact, in assessing 10-year survival following curative hepatectomy, Tomlinson et al examined 612 patients of which 25% presented with bilobar disease and investigated multiple preoperative prognostic factors to assess survival and found that bilobar disease did not preclude long-term survival and cure [3].

Bolton et al reported on 121 patients with CLM who underwent "simple" resections compared to 44 who had "complex" resections of which 98% had bilobar metastases [28]. Complex resections did not result in significantly higher perioperative mortality compared to simple resections (9% vs. 5%, respectively) nor did they significantly reduce five-year OS (37%, median 39 ± 11 months versus 36%, median 43 ± 4 months, respectively) suggesting that an aggressive or "complex" approach is reasonable in well selected patients.

Fong et al. reviewed 1001 patients who underwent hepatectomy for CLM of which 40% were noted to have bilobar disease [13]. Though, bilobar disease did represent an adverse predictor of outcome compared to unilobar disease with five-year survivals of 29% and 38%(p = 0.02), respectively, bilobar disease was not a predictor of poor outcome and recurrence by multivariate analysis as opposed to the five factors associated with Fong's CRS discussed previously. Hence, the CRS used for prognosis of outcome and patient selection does not include bilobar disease and survivals are favorable to justify pursuing a surgical approach in highly selected patients with bilobar disease.

## Technical Considerations

At the heart of the expansion of indications for hepatectomy for CLM has been a decrease in the perioperative morbidity and mortality associated with major hepatectomy in the modern era. Over the last two decades, we have seen improvement in surgical techniques to optimize the safety and outcomes of surgical resection. Better understanding of the internal anatomy of the liver as described by Couinaud in the 1950's clarified segmental hepatic anatomy. This knowledge, combined with the use of intraoperative ultrasound, has allowed for intraoperative mapping of the vascular anatomy and tumor relationships to optimally plan surgical resections, thus minimizing morbidity and mortality. Other important components to minimize the mortality and morbidity of hepatic resection include the use of low central venous pressure(CVP) anesthesia. Melendez et al. examined this concept in the 1990's in which 496 patients underwent low CVP hepatic resections [29]. There were no intraoperative deaths with perioperative mortality rate of 3.8%, median blood loss of 645 mL, and no transfusions necessary in 67% of patients. Low CVP allowed safe resection by minimizing blood loss and mortality without detrimental effects on renal or hepatic function and has been further corroborated in other studies [30,31].

### Synchronous Disease

The management of the primary tumor and synchronous liver metastases remains an interesting challenge. Various approaches have been described including concomitant primary and metastases resection, as well as staged approaches.

There has been historical concern regarding synchronous resections of colorectal primaries and the hepatic metastases with mortalities as high as 17% being reported [28]. As a result, there has been apprehension to this approach prompting multiple studies examining the safety of a synchronous versus staged approach.

The largest study, by Reddy et al., examined 610 patients with stage IV CRC with CLM, of which 135 underwent concomitant hepatectomy and primary colorectal resections and 475 underwent staged resections [32]. Simultaneous resection was associated with fewer (median 1 versus 2, p = 0.01) and smaller (median 2.5 versus 3.5 cm, p = < 0.0001) metastases. Fewer simultaneous resection patients underwent major (≥ 3 segments) hepatectomies compared to staged resections(26.7% versus 61.3%, p < 0.05). Hospital stay was shorter after simultaneous resections compared to the cumulative hospitalization for staged resections (median 8.5 vs. 14 days, p < 0.0001). Importantly, the mortality (1.0% versus 0.5% for simultaneous and staged, respectively) and severe morbidity (14.1% versus 12.5% for simultaneous and staged, respectively) were similar after simultaneous colorectal resection with minor hepatectomy compared to minor hepatectomy alone (both p > 0.05). This suggests that the colon resection did not contribute adversely to outcome. However, with major hepatectomy, comparisons of simultaneous colorectal resection to staged resection patients(does not include patients with staged resections at different institutions) resulted in increased mortality (8.3% versus 1.4%, p < 0.05) and severe morbidity (36.1% versus 15.1%, p < 0.05). Reddy et al. also addressed the confounding variable of different institutions in an additional sub-analysis examining patients undergoing major hepatectomy with simultaneous resection demonstrating greater severe morbidity compared to single institution staged resections(36.1% versus 17.6%, p = 0.05), but was similar among patients undergoing minor hepatectomy (14.1% versus 10.5%, p > 0.05). Hence, major hepatectomy appears to independently predict severe morbidity after simultaneous resections [hazard ratio (HR) = 3.4, p = 0.008]. The validity of this data may be questioned secondary to the fact that only 14.7% of staged patients underwent staged resections at a single institution. As such the reported morbidity may underestimate the associated total morbidity secondary to inadequate data capture. The authors concluded that synchronous resection with simultaneous minor hepatectomy is safe with shorter hospital stay. However, synchronous resection with major hepatectomy should be performed only in highly selected patients so as to minimize associated morbidity and mortality.

More recently, Martin et al. examined 230 patients, of which 70 underwent simultaneous colorectal/hepatic resection versus 160 patients who underwent staged resections [33]. In this study, simultaneous versus staged operations were similar for major hepatic resections performed(≥ 3 Couinaud segments)(32% vs. 33%), size of hepatic metastases (4 cm vs. 3.7 cm), and number of hepatic metastases(3 vs. 3), respectively. Complication rates and severity were also similar between groups(55% vs. 56%), respectively. As may be expected, the simultaneous group had a shorter length of stay compared to the cumulative length of stay in the staged group (10 days vs. 18 days, respectively, p = 0.001). The authors concluded that simultaneous resection remains an acceptable option for those patients with synchronous disease with similar mortality and morbidity and a decreased length of hospital stay.

When considering a staged approach to synchronous CLM, the order of resection is worthy of discussion. Traditionally, the primary tumor has been addressed initially followed by treatment of liver disease. There has been historical concern regarding the potential complications associated with the primary tumor including perforation, bleeding, and obstruction that led to a dogmatic primary-first approach followed by management of liver disease. Increasingly, a liver first approach has been explored.

Scoggins et al. retrospectively examined 89 patients with synchronous CLM to determine the impact of the management of the primary tumor on morbidity and survival [34]. Sixty-six patients underwent resection of the primary, while 23 patients with an asymptomatic primary received chemotherapy, external beam radiotherapy or combined chemoradiation. The median survival was similar between those who underwent surgical and non-surgical management of their primary(14.5 months vs. 16.6 months, respectively, p = 0.059). The operative group had a perioperative morbidity rate of 30.3% and mortality rate of 4.6%. The nonoperative group had a surgery-free survival of 91.3% with two patients(8.7%) ultimately requiring emergent diversion secondary to obstruction. No complications of perforation or bleeding occurred in the nonoperative group. This study suggests that it is not necessary to first address the primary tumor prior to intervention for CLM.

Mentha et al. corroborates a liver first approach in which they treated 35 patients with chemotherapy initially followed by liver resection and then treatment of the primary tumor [35]. Five patients were unable to complete treatment (one chemotherapy-related sepsis, one complete response of liver metastases, three with progression). Three- and five-year survival rates of the remaining 30 patients were 60% and 31%, respectively with overall median survival of 44 months. This approach was also supported in other studies by Poultsides et al. and Puthillath et al in which asymptomatic patients with stage IV CRC were safely treated with up front chemotherapy and deferral of surgery [36,37]. Hence, it is reasonable to pursue a liver-first approach in patients with asymptomatic primary tumors using nonoperative management of the primary with low risk of complications and no statistically significant adverse effect on survival, while minimizing the risk of CLM disease progression during treatment of the primary.

### Portal Vein Embolization

Paramount in devising hepatectomy to allow complete extirpation of all disease is leaving adequate FLR to prevent postoperative hepatic insufficiency. In patients with an anticipated inadequate FLR or when regeneration is likely to be impaired, such as in the face of cirrhosis or chemotherapy-induced steatohepatitis, initiation of the regeneration process prior to hepatectomy potentially reduces the risk of postoperative hepatic insufficiency. Portal vein embolization (PVE) was first described in 1990 by Makuuchi as a means to hypertrophy the FLR prior to liver resection [38]. This approach employs preoperative embolization of the portal vein to all tumor-bearing liver, thus allowing hypertrophy of the contralateral FLR [39]. It is necessary to assess the FLR relative to total liver volume to determine the need for PVE. The FLR(%) may be calculated by employing radiographically obtained FLR volumetry, as well as established formulas for total liver volume(TLV) and body surface area(BSA). The FLR(%) is calculated using the formula: FLR(%) = FLR(radiographic volumetry, cm^2^)/TLV. The FLR(volumetry) is determined by obtaining an estimated radiographic absolute volume(cm^2^) of the FLR using computed tomography(CT) with three dimensional reconstructions(3-D). The total liver volume is obtained using the formula: TLV(cm^3^) = 706 + BSA(m^2^) + 2.4 [40]. BSA may be calculated using the Mosteller formula: BSA(m^2^) = ([Height(cm) × Weight(kg)]/3600)^1/2^. In those patients with normal underlying liver where the FLR is anticipated to be <20%, PVE prior to extended hepatectomy potentially minimizes postoperative morbidity. In patients with cirrhotic/fibrotic livers, as well as those who have had extensive chemotherapy where the FLR is <30%, PVE should also be considered [41].

**Figure 1:**
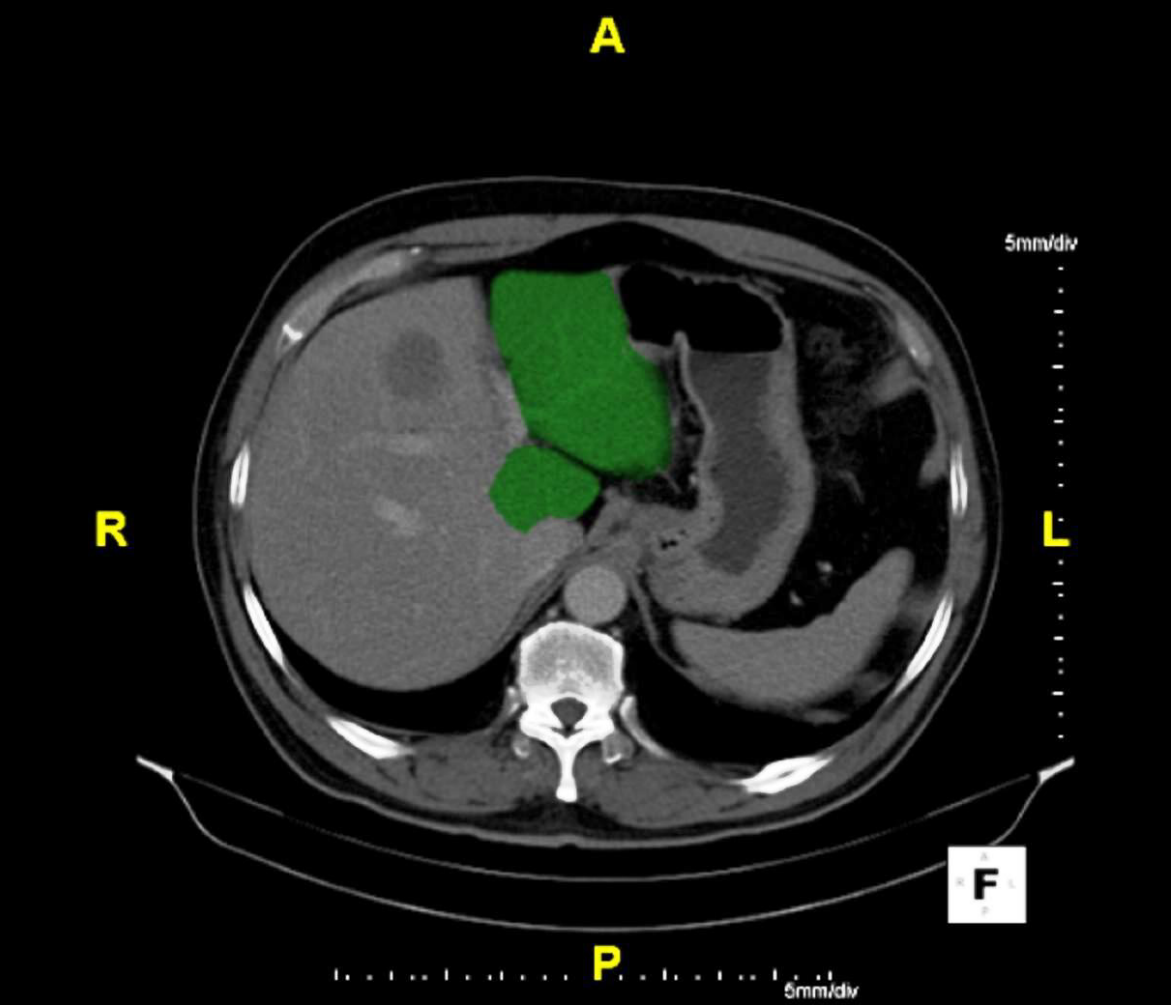
**CLM with Small FLR Prior to Hepatic Resection**. Patient with metastatic rectal carcinoma with right hepatic metastasis who was an operable candidate for right hepatectomy, but on preoperative computed tomography(CT; 5/12/09), the patient had a small left hepatic lobe and FLR.

**Figure 2:**
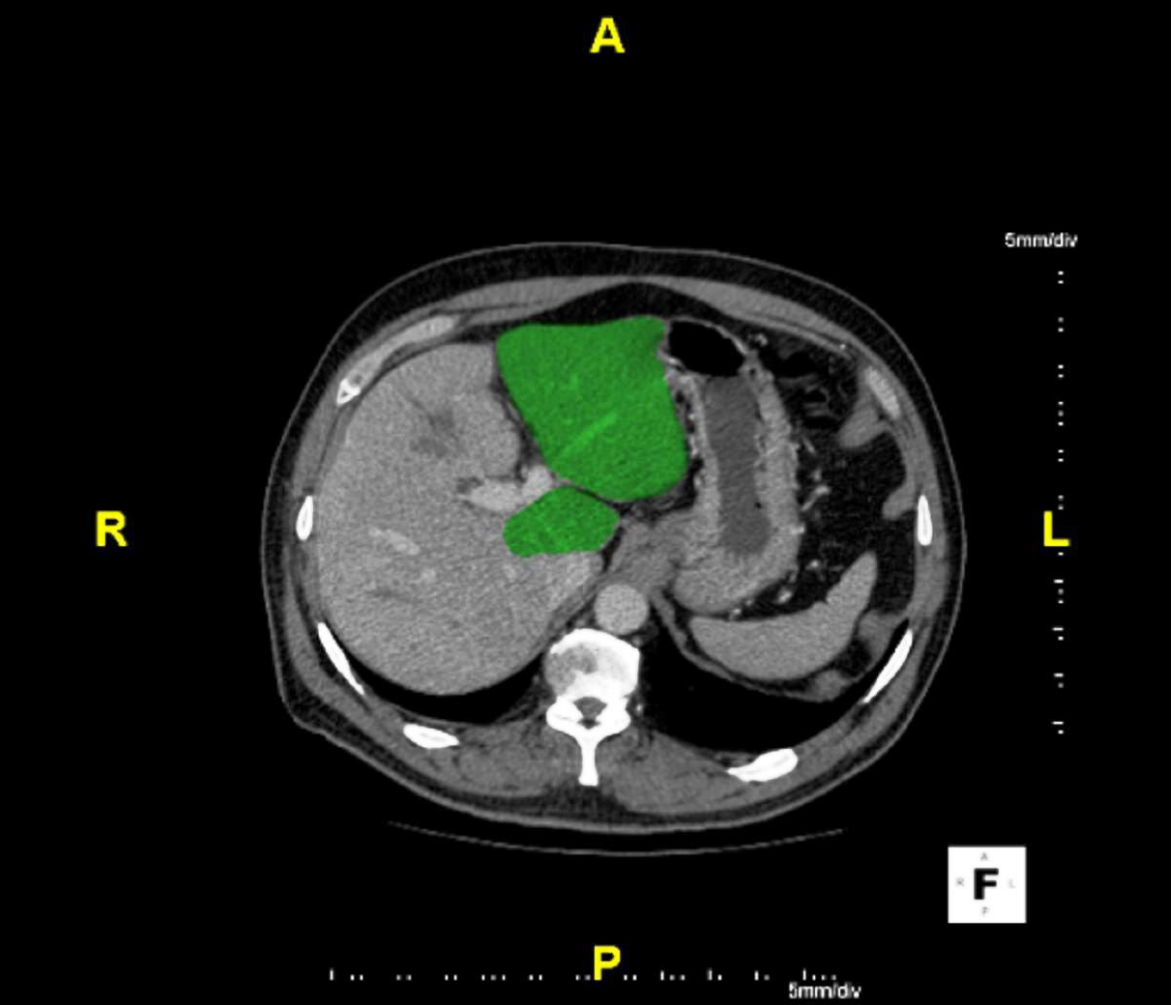
**CLM after PVE for Small FLR Prior to Hepatic Resection**. Patient(as in Figure 1) with metastatic rectal carcinoma and right hepatic metastasis who underwent right PVE(6/10/09) with followup CT(7/13/09) with demonstrable growth of the FLR.

**Figure 3:**
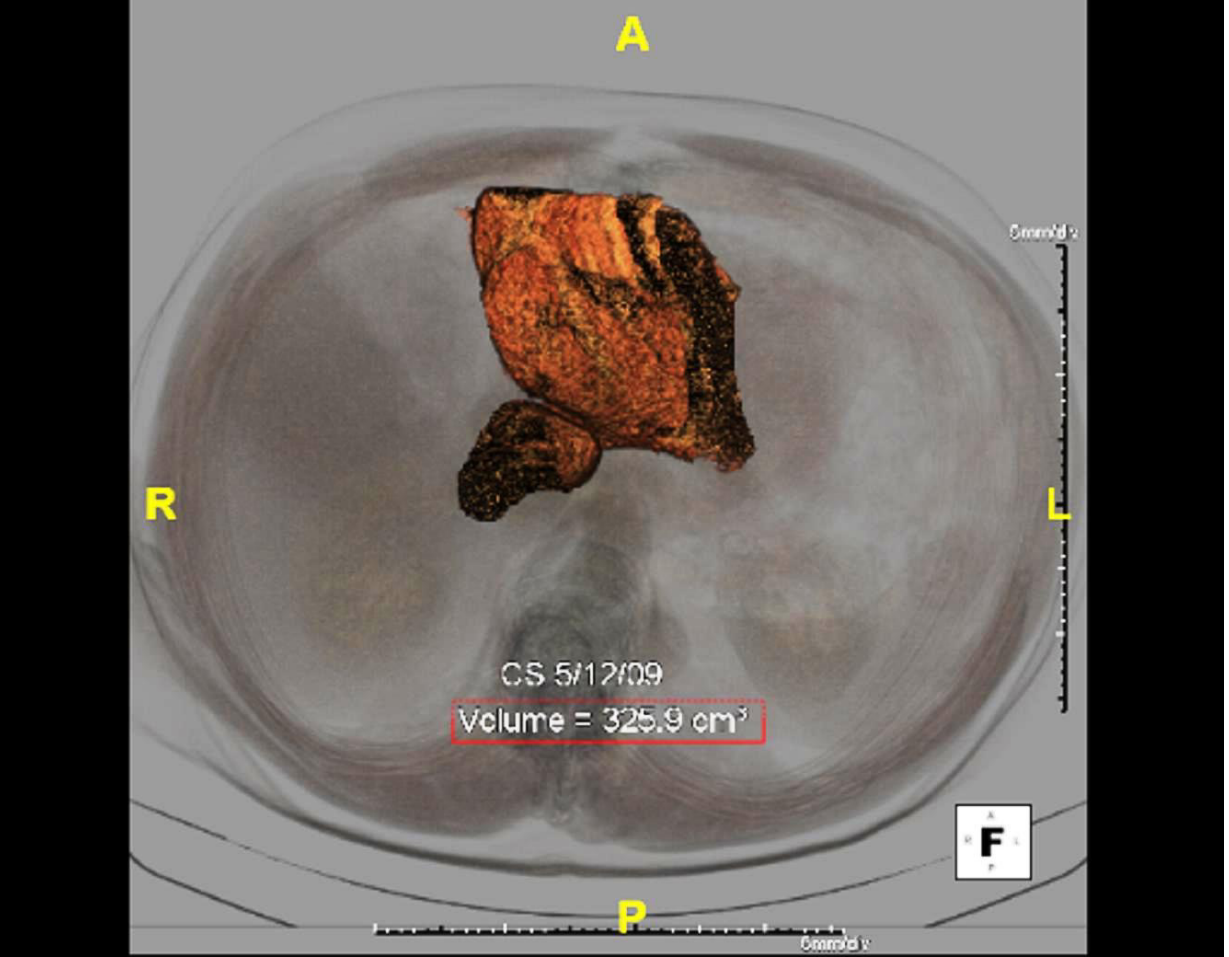
**Three Dimensional(3-D) Reconstruction of Small FLR with calculated volume measurements**. Patient(as in Figure 1, 2) with metastatic rectal carcinoma and right hepatic metastasis with small FLR with radiologically calculated volume of 325.9 cm^2^. Using standardized volumetric formulas, this patient had a FLR of approximately 19%.

**Figure 4:**
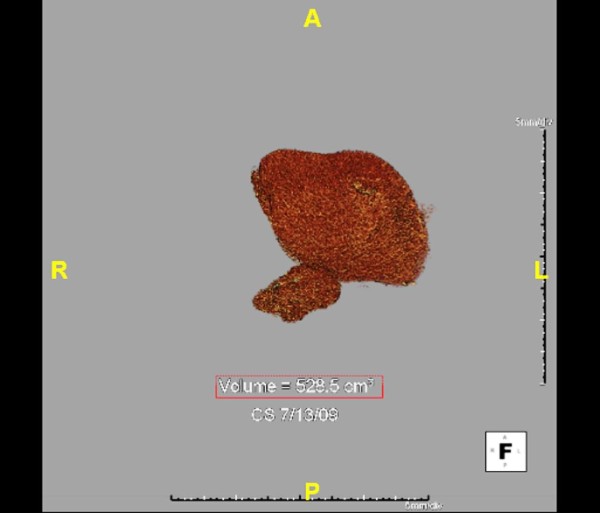
**3-D Reconstruction of FLR after Right PVE for Small FLR**. Patient(as in Figures 1, 2, and 3) with metastatic rectal carcinoma and right hepatic metastasis with small FLR(19%) that underwent right PVE with good response and growth of left hepatic lobe with calculated FLR volume of 528.5 cm^2^. The FLR after PVE was approximately 31%.

PVE has been a useful adjunct to increase the pool of potential resection candidates. Abdalla et al. examined 42 patients planned for extended right hepatectomy [42]. Groups were divided into 18 patients that underwent PVE compared to 24 who did not. In normal circumstances, patients subjected to PVE would not have been offered resection for fear of postoperative liver failure. In those undergoing preoperative PVE, the FLR increased from 18% to 25%. No significant difference between groups was seen for morbidity and mortality. The overall three-year survival was 65% with statistically similar median survival of 40 vs. 52 months for PVE and no PVE, respectively. As a result, PVE enabled safe and potentially curable resection in patients who were otherwise not deemed surgical candidates. Whether there is any oncologic effect of preoperative PVE is yet to be determined. Current recommendations are for PVE to be undertaken in patients with otherwise normal livers when FLR is less than 20%, in patients with steatohepatitis or steatosis due to chemotherapy or other etiology when FLR is less than 30%, and in patients with severe fibrosis or cirrhosis when FLR is less than 40%. An increase in FLR can be seen 4-6 weeks following PVE suggesting that the liver is capable of regeneration and has typically attained adequate volume to proceed with surgical intervention with the hope of minimizing postoperative hepatic insufficiency. This is well illustrated in examining the FLR growth from figure 1 to figure 2. Additional radiographic 3-D reconstructions in figure 3 and 4 demonstrate calculated FLR volumes and illustrate the growth after PVE that may be achieved prior to hepatic resection

### Two-Stage Hepatectomy

Patients with CLM have traditionally been classified as "resectable" or "unresectable" largely based on technical considerations. Unresectable patients with bilobar disease are not safely able to have their tumors completely resected at one operation, even in patients with low volume tumor burden. However, a third group of patients has now arisen, the "sub-optimally resectable." These patients typically have multifocal bilobar disease in which the management of these patients is complex. These patients often receive chemotherapy given the extent of disease and this is valuable in patient selection because chemotherapy may aid in downsizing tumor burden as well as determine patient tumor biology that would in fact benefit from an aggressive surgical approach. With adjuvants to surgery including ablative therapy and PVE, different strategies have been devised to achieve extirpation multifocal bilobar disease using a two-stage approach. One of the major questions with regards to two-stage hepatectomy is how and in what order to address bilobar disease.

In an early series, Adam et al first described their experience with staged hepatectomy in 16 patients with "unresectable" bilobar CLM [43]. The hepatic tumor burden is typically more predominant on the right side. Hence, the initial operation consisted of a right hepatectomy addressing the more significant tumor burden and without incidence of perioperative mortality. Following left liver regeneration after the initial resection, 13 patients underwent repeat resection for the residual left sided disease, eight of which were major hepatectomies with two resulting perioperative deaths. Three patients did not undergo a second stage resection secondary to disease progression between procedures, one with intrahepatic progression and two with extrahepatic progression. All patients received chemotherapy before and after hepatectomy. Median OS was 31 months from the second hepatectomy and 44 months from the initial diagnosis of liver metastases. Four patients developed hepatic recurrence, of which three underwent a third resection. This early work examining two stage hepatectomy demonstrated need for careful surgical planning with inherent risk of morbidity and mortality in these patients. Theoretical advantage to this approach is that at the initial stage, a majority of tumor burden is addressed with benefit of FLR regeneration. However, one must consider that this approach has a more extensive initial operation resulting in more tedious and difficult dissection at the time of the second stage operation. Additionally, there is theoretic exposure of left sided metastases to growth factors associated with hepatic regeneration and could lead to increased growth rate of the existing tumor burden.

Alternatively, Adam et al. further examined a staged approach in which the left sided hepatic tumor burden was addressed initially and occasionally in conjunction with a right PVE [44]. PVE may be an important adjunct in patients with bilobar disease requiring two stage approach to promote hepatic regeneration of the FLR when necessary. PVE allows for hypertrophy of the FLR in which the disease has been cleared, thus allowing for a second stage procedure (e.g. hemihepatectomy or extended hepatectomy) in a reported 75.7% of patients resulting in one- and three-year survivals of 70% and 54.4%, respectively [45,46]. Following regeneration of the left lobe when right PVE was performed, a right hepatectomy was then performed to complete extirpation of hepatic disease. Obviously, in this approach, patients must have a limited tumor burden on the left requiring limited resection in order to allow for an adequate FLR and subsequent regeneration. PVE was employed in 20 patients to aid in hepatic regeneration. Adam et al. had 31/45(69%) patients completed the planned two stage hepatectomy in which 23 underwent left side initial approach and the remaining 8 patients underwent right hepatectomy initial approach. Multiple studies demonstrated completion of two stage approach ranging from 76%-100% and second stage procedures often deferred secondary to disease progression [44,45,47-49]. In comparing both approaches, Adam et al. found no significant difference in complications, operative times, or blood loss. The second stage procedure carried greater associated morbidity and mortality. General complications were 6.5% and 22.6% for first and second stage, respectively. Likewise, liver related complications were 12.9% and 48.4% for first and second stage, respectively. Second stage procedures carried longer operative time, increased blood loss, hospital stay, vascular clamping time. Two(6.5%) perioperative mortalities were seen after the second operation. It appears evident that hepatic reoperation carries significant morbidity and mortality regardless of approach. However, regardless of approach, favorable outcomes were obtained with actuarial 3-year and 5-year survival of 47% and 28%, respectively with median survival of 35 months. Fifteen(48.4%) patients developed hepatic recurrence of which eight patients underwent reoperation. A left sided first approach facilitates clearance of left sided hepatic disease prior to PVE thereby eliminating the theoretical exposure of tumor to growth factors that may perpetuate growth after a PVE. Additionally, an initial procedure addressing left sided hepatic disease will likely require less extensive dissection and thereby facilitate an easier dissection at the time of a second, often more involved operation.

Jaeck et al also examined 33 patients with "unresectable" CLM in which major hepatectomy was undertaken at the second operation five to eight weeks following a minor hepatectomy combined with interval right PVE(± Couinaud segment IV) was performed [45]. Second hepatectomy was able to be accomplished in 28 patients with no perioperative mortality. Five patients had disease progression prior to the second operation. Three-year OS following two-stage hepatectomy was 54%.

With the improvements in modern day chemotherapy, two-stage hepatectomy has become a viable option for those patients that were previously considered "unresectable" for multifocal, bilobar disease. Now patients that are responsive to chemotherapy may be considered "sub-optimally resectable" and should be considered for an aggressive surgical approach in a multidisciplinary tertiary care setting.

## The Impact of Chemotherapy on Hepatectomy

Chemotherapy may have had the biggest impact on the approach to management of CLM. A decade ago, chemotherapy options were limited to traditional therapy with fluorouracil(5-FU) and leucovorin. With the emergence of newer cytotoxic(e.g. oxaliplatin, irinotecan) and biologic(epidermal growth factor receptor(EGFR) and vascular endothelial growth factor(VEGF) inhibitors) agents, multiple options are now available. The timing of chemotherapy has been debated as to whether neoadjuvant, perioperative, or adjuvant therapy is optimal for those patients with CLM.

Alberts et al. examined 42 patients with unresectable CLM treated with a median six months of FOLFOX4 neoadjuvant chemotherapy. Radiologic response was seen in 25(60%) patients including one complete response. Following response to therapy, 17(40%) patients proceeded to surgery with 14 patients able to have complete resection, one with partial resection, and two still being considered unresectable. This conversion to resectable disease resulted in a median survival of 26 months [50].

Recently, the European Organization for Research and Treatment of Cancer (EORTC; Nordlinger et al.) asserted there is potential benefit to the use of perioperative chemotherapy in those patients who are initially resectable demonstrating increased progression-free survival(PFS) over those patients who underwent surgery alone (35.4% vs. 28.1%, respectively; p = 0.058) [51]. Additionally, in another study by Nordlinger et al.(Eastern Cooperative Oncology Group - ECOG), they examined neoadjuvant chemotherapy and its use in all patients regardless of initial resectability status because it improved complete resection rate, limited extent of resection, assessment of tumor biology and response to chemotherapy. Four other studies support the conclusions of the EORTC study supporting the use of chemotherapy as a bridge to resectable disease in the subset of patients who are initially unresectable [51-55].

With the improvement in response rates, survival, and the ability to downsize unresectable CLM with modern day chemotherapy, it has become necessary to also acknowledge the associated toxicities when employing them as a part of a multimodality approach [56-60]. With oxaliplatin treatment, there has been demonstration of hepatic sinusoidal obstruction that increases with prolonged treatment(i.e. greater than six cycles) [59]. The sinusoidal obstruction syndrome(SOS) is a veno-occlusive phenomenon that has historically been associated with bone marrow transplantation. However, as oxaliplatin use has became more prevalent, there is note of increased association with SOS compared to other chemotherapy regimens. Vauthey et al. demonstrated sinusoidal dilatation with oxaliplatin that was five times more than that with irinotecan therapy(19 vs. 4%, respectively) [57]. Grossly, this is demonstrated by a 'blue liver' that is secondary to trapped erythrocytes within the hepatic sinusoids. Vauthey et al. also demonstrated an association between irinotecan and steatohepatitis [57,59]. The development of steatohepatitis is significant because it has been associated with hepatic insufficiency and failure, as well as death after liver resection. Bevacizumab has demonstrated efficacy at decreasing the oxaliplatin associated sinusoidal injury and has demonstrated safety when discontinued greater than 5 weeks prior to surgery [59]. However, bevacizumab has also been associated with gastrointestinal perforations and bleeding complications [61,62]. There has been additional concern regarding liver regeneration after resection with the use of bevacizumab in those patients undergoing PVE. However, it would appear that bevacizumab does not significantly alter the hypertrophy response of the liver following PVE [63]. As chemotherapy regimen options expanded, it has become imperative to consider each regimen's associated toxicities to best plan the regimen type and duration of therapy so as to minimize toxicity. Hence, a multidisciplinary approach with medical oncology and surgeons in a tertiary care institution may be optimal for patient outcomes in light of the considerations of chemotherapy, associated toxicities, and timing of surgical intervention.

## Non-Resectional Locoregional Therapy

### Thermal ablation

Local therapy options use targeted energy to destroy tumor cells. Such thermal ablation can be done by cooling, in the case of cryoablation, or by heating tumor with high-energy radio waves. Given the wider use of the latter, we will focus our discussion on radiofrequency ablation (RFA). The goal of RFA is to heat tumoral tissue and a margin of surrounding normal parenchyma to a point that induces coagulative necrosis. This is done by insertion of a needle-type electrode into tumor tissue with an alternating current that is generated between the implanted electrode and a dispersive skin electrode/grounding pad, resulting in ionic agitation, frictional heating, and ultimately coagulation necrosis of the tumor tissue [64]. This can be completed percutaneously, as an open surgical procedure, or laparoscopically and has been increasingly used for patients that are high surgical risk or in situations where preservation of normal hepatic parenchyma is paramount. Multiple studies have attempted to compare ablation to resection for a variety of hepatic tumors, particularly CLM, to define its role in the treatment algorithm. Most studies focus on local recurrence rates, and have demonstrated wide variability from 1.7% to over 66.7% [65-72].

Several technical factors account for this wide variance in local recurrence rates. These include tumoral factors, operator factors, and technical factors. Consistent throughout the literature has been the impact of tumor size on local recurrence rates. In a large retrospective study by Siperstein et al., 234 patients that underwent 292 ablations were examined [73]. RFA of tumors >3 cm. resulted in local recurrence rates of 28% while tumors ≤ 3 cm. recurred locally in 20% (p = 0.07). Median survival for those with >3 lesions was 17 months versus 27 months in those patients with ≤ 3 lesions(p = 0.0018). Others have demonstrated a similar negative impact of increasing tumor size on the rate of local recurrence [74-78].

Similarly, local recurrence rates are influenced by approach, with percutaneous approaches often being associated with higher recurrence rates compared to laparoscopic or open approaches [79-81]. Proximity to large vessels resulting in a "heat sink" is another limitation of ablation. The passage of blood through a large vessel in a region to be ablated produces a local cooling effect below the target temperature of 100°C. Perhaps more important is the "energy sink" that results from the travel of electric current from the energy source through the less resistant blood vessel back toward the grounding pad, thus reducing the amount of energy delivered to the tumor [82,83].

In a study by Abdalla et al., they examined 418 patients with curative intent for CLM, RFA was utilized as the sole treatment in 57, while RFA was combined with resection in 101 [71]. The remaining patients underwent resection alone (N = 190) or no local therapy (N = 70). RFA alone resulted in the highest rates of overall recurrence at any site (84%), recurrence within the liver (44%), or true local recurrence (9%) compared to recurrence rate of resection alone (52%, 11%, 2%, respectively) or recurrence rates for resection in combination with RFA (64% and 5% for overall and local recurrence, respectively). In patients with single lesions, resection resulted in a significant survival advantage over RFA alone. Noteworthy was that in spite of the inferiority of RFA to resection, it did provide a significant survival advantage over chemotherapy alone in the 70 patients where the operation was aborted (p = 0.0017). Although this study attempted to make comparisons between patients with similar tumor burden, the retrospective design cannot take into account intraoperative decision making that potentially biases the data in favor of resection. This underscores the need for prospective studies to address this issue.

The next generation of ablation techniques attempts to address many of the technical limitations described above. Microwave ablation, as its names suggests, radiates microwaves at 915 MHz from the active port of an antenna placed within a tumor via a percutaneous, laparoscopic, or open approach. While the technology works to minimize the "heat sink" effect, it too is limited by many of the same principles as RFA. Ianniti et al. examined this technology with various liver tumors in 87 patients. They undertook 94 ablation procedures in 87 patients for 224 tumors with local site recurrence of 2.7% and regional recurrence in 43% [84]. Other ablative techniques are on the horizon but are, as of yet, unproven in CLM including 2.4 GHz microwave ablation, irreversible electroporation and laser induced thermotherapy(LITT) which utilizes photons from low intensity lasers(diode, N-Yag) that are then absorbed by chromophores within cells and then converted to heat to induce tumor ablation [85,86].

### Regional Therapies

Patients with multifocal CLM who are unfit for surgery or have tumor burden/distribution not amenable to resection or ablation are potentially candidates for liver-directed therapy. Such regional therapy options include transarterial chemoembolization (TACE), radioembolization or selective internal radiation therapy(SIRT), and hepatic artery infusion pump (HAIP).

HAIP is a technology that allows for regional delivery traditionally of high dose floxuridine(FUDR) as well as other chemotherapy regimens into CLM. Fahy et al. reviewed multiple trials studying HAIP as neoadjuvant or adjuvant chemotherapy [87]. That review highlighted the limitations of multiple small trials with variable chemotherapy regimens employed in a neoadjuvant setting with response rates ranging from 16-82% with variable rates of conversion from unresectable to resectable disease from 3-47%. Thus, it remains unclear as to the efficacy of HAIP until a large, randomized trial is performed.

In the adjuvant setting, trials suggest an improved disease free survival (DFS) with the use HAIP chemotherapy. Kemeny et al. examined adjuvant HAIP chemotherapy with systemic(combined) versus systemic therapy alone(monotherapy) with encouraging results [88]. With median follow up of 10.3 years, combined therapy resulted in an increased PFS of 31.3 months compared to 17.2 months with monotherapy(p = 0.02). As well, median hepatic PFS was not yet reached in the combined group compared to 32.5 months in the monotherapy group (p < 0.01). With combined therapy, the median OS was 68.4 months compared to 58.8 months in the monotherapy group(p = 0.10), there appeared to be improved long-term outcome with 10-year survival rates of 38.7% and 16.3%, respectively. Hence, this suggests that the use of HAIP as an adjuvant chemotherapy does confer a benefit in PFS, survival free of liver progression, and potential benefit to long-term outcome.

It is important to remember that the use of HAIP does have inherent associated risks and complications. HAIP requires an operation to place the catheter within the common hepatic artery. With that, there is a moderate complication rate of which early complications are known to include extrahepatic or incomplete perfusion, gastritis, ulcers, arterial/catheter thrombosis, and infection. Late complications include hemorrhage and biliary sclerosis.

Selective internal radiation therapy (SIRT) has been a fairly recent technology that has been used for the regional delivery of radioactive beads simultaneously to multiple tumors. Millions of microscopic radioactive spheres are delivered via a catheter placed in the femoral artery into the hepatic artery. Each sphere (approximately 35 microns in size) is bonded to yttrium-90^(90^Y^) ^which is a beta particle emitter with a half-life of 64 hours and penetration of only two to three millimeters. These beads are small enough to penetrate the microvasculature of the tumors but large enough to lodge in the precapillary arterioles. Slow decay of the ^90^Y results in tumoral radiation as high as 150-200 Gy with relatively little radiation (i.e. <45 Gy) to normal hepatic parenchyma.

Candidates for SIRT are considered if they have good hepatic reserve, patency of the main portal vein, and less than 10% hepato-pulmonary shunting of blood as determined by a pre-procedure tagged macroaggregated albumin (Tc^99^-MAA) scan. Hepatic arteriogram is first undertaken with embolization of visceral vessels as needed to prevent microsphere spread to extrahepatic normal tissues. Hepato-pulmonary shunting >20% prohibits SIRT due to risk of radiation pneumonitis whereas shunting between 10-20% requires reduction in calculated dosimetry, and <10% is safe to proceed. Rare complications include radiation induced liver disease, gastrointestinal ulceration, and hemorrhage [89-91].

SIRT is still a novel therapy being employed so there are limited studies at this point examining its efficacy, response, and toxicity. Kennedy et al. examined 208 patients that received salvage therapy due to CLM refractory to oxaliplatin and irinotecan therapy [92]. This study found that those patients who responded had median survival of 10.5 months versus 4.5 months in nonresponders without any procedure related deaths or liver failure. Partial radiographic response was noted by CT scan in 35% and by PET scan in 91% with a biochemical response in CEA reduction seen in 70%.

Four prospective trials of SIRT for CLM are completed or ongoing [90]. A phase III trial examining SIRT + hepatic artery chemotherapy (HAC) vs. HAC alone was conducted in 74 patients. The complete response(CR) and partial response(PR) and PFS was increased for combination therapy (44% and 15.9 months) versus HAC alone (17.6% and 9.7 months)(p = 0.01) suggestive of a survival benefit for the addition of SIRT.

A small randomized phase II/III trial examining the use of SIRT and 5-FU/leucovorin versus chemotherapy alone in 21 patients yielded promising results [90]. The combination therapy demonstrated increased PFS (18.6 vs. 3.4 months, p < 0.001) and OS(29.4 versus 12.8 months, p = 0.02) compared to chemotherapy alone.

A small phase I trial from Australia and the United Kingdom by Sharma et al. examined 20 unresectable, chemotherapy-naive CLM patients using FOLFOX4 and SIRT with dose escalation of oxaliplatin to standard 85 mg/m^2^[93]. Median PFS was 9.3 months and median time to hepatic progression was 12.3 months. Two patients proceeded to partial liver resection.

An additional dose escalation phase I trial by Goldstein et al. examined combination therapy with SIRT and irinotecan in 25 irinotecan-naïve patients who had failed chemotherapy previously [94]. Dose escalation to 100 mg/m^2 ^with SIRT was administered as tolerated with acceptable toxicity. Results demonstrated 9/17 patients with partial response, with median time to hepatic progression of 7.5 months and median OS of 12 months.

Stubbs et al. examined 50 patients with advanced, unresectable colorectal liver metastases and SIRT [95]. Patients tolerated SIRT with no associated mortality but with some treatment associated morbidity, most notably a 12% duodenal ulceration rate. Serial CEA levels and radiographic response were followed. Median CEA at 1 and 2 months post-treatment were 19 and 12% of baseline, respectively. A statistically significant survival benefit was seen in those patients without extrahepatic disease in which they had median survival of 17.5 months with estimated survival of 79.2%, 66.7%, 55.9%, 25.2%, and 16.8% at 6, 12, 18, 24, and 30 months, respectively when compared to those patients with extrahepatic disease. Tumor marker decline was observed in more than 90% of patients after a single treatment and survival times, particularly for those who did not develop extrahepatic metastases for some time, appear to be prolonged.

In an additional study, Stubbs et al. examined 100 patients with advanced, unresectable colorectal liver metastases [96]. SIRT was performed and median CEA levels three months after treatment were 18% of the original baseline CEA level. Radiographically, only 5/80(6.25%) patients demonstrated progression of disease. Patients with tumor marker response demonstrated increased survival as well suggesting a benefit of SIRT in this patient population.

## Conclusion

The management of CLM has evolved as therapeutic options have continued to expand and grow. Traditional dogma that had once previously limited treatment for the most part is no longer valid. Many characteristics that used to be considered contraindications to surgical intervention are no longer substantiated. Modern cytotoxic chemotherapy along with addition of biologic agents has generated improvement in response, survival, and the feasibility of offering patients various surgical and locoregional interventions. As surgical intervention has been made more feasible by modern day chemotherapy, improved surgical techniques and adjuncts to resection(e.g. RFA, PVE) has facilitated more patients being offered potentially curative therapy. Additionally, locoregional therapies including TACE, SIRT, and HAIP remain reasonable options for high risk patients and those with unresectable disease. On the whole, the management of CLM has seen great strides made and we continue to evolve the therapeutic options and approaches with improvement in patient outcomes. Therefore, in order to provide optimal outcomes for patients with CLM, it is imperative to consider all treatment options available based on each patient's clinical picture and best care may be undertaken in a tertiary care center where a multidisciplinary approach can be offered and pursued with collaboration of surgeons, medical oncologists, and interventional radiologists.

## Abbreviations

AHPBA: American Hepato-pancreatico-biliary Association; BSA: Body Surface Area; CEA: Carcinoembryonic Antigen; CLM: Colorectal Liver Metastasis(es); CRC: Colorectal Cancer; CRS: Clinical Risk Score; CVP: Central Venous Pressure; DFS: Disease Free Survival; ECOG: Eastern Cooperative Oncology Group; EORTC: European Organisation for Research and Treatment of Cancer; EHD: Extrahepatic Disease; FLR: Future Liver Remnant; FUDR: Floxuridine; HAIP: Hepatic Artery Infusion Pump; LITT: Laser Induced Thermotherapy; OS: Overall Survival; PFS: Progression Free Survival; PVE: Portal Vein Embolization; RFA: Radiofrequency Ablation; SIRT: Selective Internal Radiation Therapy; SOS: Sinusoidal Obstruction Syndrome; SSAT: Society for Surgery of the Alimentary Tract; SSO: Society of Surgical Oncology; TACE: Transarterial Chemoembolization; TLV: Total Liver Volume; ^90^Y: Yttrium-90.

## Competing interests

The authors declare that they have no competing interests.

## Authors' contributions

SA, PMB, and CS collaborated on the format and structure of this review paper. SA was primary author of this review. PMB and CS provided critical analysis and assisted in editing of the review through all stages of drafting. All authors read and reviewed final draft of this manuscript.
